# Tumor-Associated Macrophages and Ovarian Cancer: Implications for Therapy

**DOI:** 10.3390/cancers14092220

**Published:** 2022-04-29

**Authors:** David Schweer, Annabel McAtee, Khaga Neupane, Christopher Richards, Frederick Ueland, Jill Kolesar

**Affiliations:** 1 Markey Cancer Center, Division of Gynecologic Oncology, University of Kentucky, Lexington, KY 40536, USA; dssc224@uky.edu (D.S.); frederick.ueland@uky.edu (F.U.); 2 School of Medicine, University of Kentucky, Lexington, KY 40536, USA; annabel.mcatee@uky.edu; 3 Department of Chemistry, College of Arts and Sciences, University of Kentucky, Lexington, KY 40536, USA; krne226@uky.edu (K.N.); chris.richards@uky.edu (C.R.); 4 Department of Pharmacology and Toxicology, University of Kentucky, Lexington, KY 40202, USA

**Keywords:** ovarian cancer, immunotherapy, macrophages, TAM, repolarization

## Abstract

**Simple Summary:**

Ovarian cancer is a highly lethal female malignancy with high rates of advanced stage and recurrent disease. Ovarian cancer is characterized as poorly responsive to immunotherapy. This is hypothesized to be secondary to its highly immunosuppressive and phenotypically “cold” tumor microenvironment (TME). Tumor-associated macrophages (TAMs) are an integral component of the ovarian cancer TME, and predominantly display an M2 protumor phenotype. Ovarian cancer cells and TAMs interact via a complex network of signaling cytokines. Ovarian cancer TAMs are linked to tumor metastasis, angiogenesis, chemoresistance, and poor prognosis. Due to their profound role, TAMs represent a therapeutic target for ovarian cancer immunotherapy. Strategies for targeting TAMs include increasing phagocytosis, decreasing recruitment, decreasing macrophage survival, and repolarizing TAMs to an antitumor phenotype.

**Abstract:**

The tumor microenvironment (TME) has been implicated to play an important role in the progression of ovarian cancer. One of the most important components of the TME is tumor associated macrophages (TAMs). Phenotypically, macrophages are broadly categorized as M1 pro-inflammatory or M2 anti-inflammatory, based on the cytokines and chemokines that they secrete. The tumor microenvironment is associated with macrophages of an M2 phenotype which suppress the surrounding immune environment, assist tumor cells in evading immune targeting, and support tumor growth and metastasis. Contrarily, M1 macrophages help mount an immune response against tumors, and are associated with a more favorable prognosis in solid tumors. One of the characteristic indicators of a poor prognosis in ovarian cancer is the overrepresentation of M2-type TAMs. As such, therapeutic modalities targeting TME and TAMs are of increasing interest. Pharmacological approaches to eliminate TAMs, include decreasing macrophage survival and recruitment and increasing phagocytosis, have been underwhelming. Clinical strategies targeting these macrophage subtypes via repolarization to an M1 antitumoral state deserve increasing attention, and may serve as a new modality for immunotherapy.

## 1. Introduction

Ovarian cancer is the deadliest gynecological malignancy in the US, with an estimated 19,880 new cases and 12,810 deaths in 2022 [1]. Most ovarian cancer cases present in an advanced stage (IIIc or higher), and the mainstay of treatment is a combination of surgery and systemic chemotherapy. Despite a high likelihood of a clinical response from primary therapy, more than 80% of advanced stage disease will recur [2,3,4]. Due to the high recurrence rate and subsequent dismal survival outcomes, there is a demonstrated need for new or modified therapies for ovarian cancer. There has been increasing recognition that epithelial ovarian cancer (the most common histopathology) is a partially immunogenic disease, and that there is a potential role for immunotherapy [5,6,7].

Macrophages encompass a significant component of the human innate immune system, and are an attractive target for new immunotherapies [8]. The primary immune system function of macrophages relies on their phagocytic function, the process of ingesting a variety of cellular substrates, mainly bacteria and cellular debris, in the setting of infection. Yet, it has been noted that macrophages play additional roles in tissue repair and development, and can exhibit considerable functional plasticity depending upon their tissue environment. Colony-stimulating factor-1 (CSF-1) and colony-stimulating factor-1 receptor (CSF-1R) are considered the major regulators of macrophages; however, several additional cytokines contribute significantly to the differentiation of macrophages to varying subpopulations with unique functional roles [9]. The most frequent sub-classification of macrophage states involves an M1/M2 paradigm, in which M1 macrophages involve a pro-inflammatory, anti-infectious state, whereas M2 macrophages exhibit an anti-inflammatory, pro-tumoral phenotype [10].

**Figure 1:**
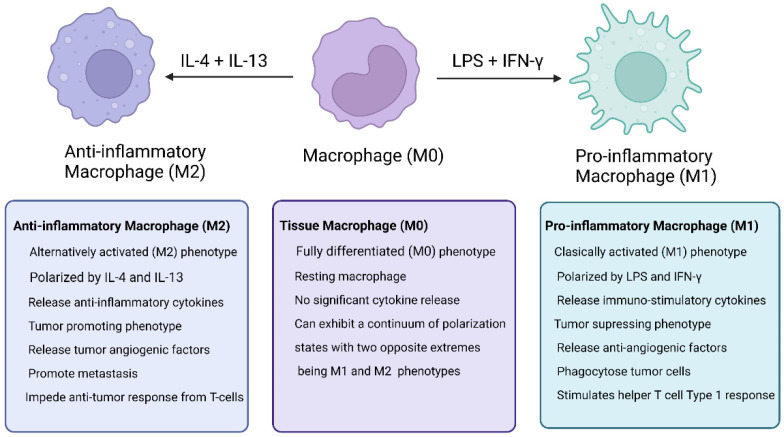
Comparative differences between M1 and M2 macrophages. Created with BioRender.com. Accessed on 21 April 2022.

Macrophages that either reside or are recruited to tumor tissue are described as tumor-associated macrophages (TAMs), and can display a wide functional phenotypic expression depending upon the microenvironment (Figure 1). TAMs appear to have a dual origin; one is via the recruitment and local differentiation of circulating monocytes from bone marrow, and the second is tissue-resident macrophage differentiation (e.g., peritoneal macrophages, brain microglia, etc.) that is seeded in resident tissues from embryonic precursors [8,9]. By volume and number alone, TAMs are the most abundant immune cell in a tumor microenvironment, and several studies have linked progression and survival to certain characteristics of the tumor microenvironment (e.g., M1:M2 ratio) [11,12]. As such, TAMs have attracted interest as both a target and source of therapeutic potential [13,14]. The goal of this article is to review the current knowledge of TAMs and macrophage polarization in regards to the disease progression and treatment of ovarian cancer. 

## 2. Tumor-Associated Macrophages (TAMs)

The tumor immunologic microenvironment has been recognized as an increasingly important aspect of cancer biology, and as a potential target for precision therapy. TAMs are significant components of tumor immunology, and despite the traditional role of macrophages as anti-tumoral and cytotoxic, TAMS have demonstrated considerable protumoral properties [8,15]. TAMs have been linked to tumor progression, metastasis, angiogenesis, and migration [13,16,17]. On a cellular level, TAMS display considerable functional plasticity, and can assume a variety of pro- or anti-inflammatory modes pending the microenvironment and associated stimuli [18,19]. TAMs express high levels of inhibitory cytokines (regulator T cells, IL-10, and TGF-β) and inflammatory cytokines. They can promote tumor growth and angiogenesis via the production of growth factors, such as VEGF and PDGF, and can also inhibit immune responses against tumors [10,12,20,21,22].

The traditional framework for understanding macrophages, and thus TAMs, is the breakdown into M1 and M2 macrophage populations. Macrophages exhibit high plasticity, and can be “activated” or “polarized” to their phenotypic profile, both in vivo and in vitro, via various cytokines, such as IFNγ, lipopolysaccharide (LPS), and others, for M1 and IL-4, IL-10, IL-13 [9,19,23,24]. Traditionally, M1 macrophages are considered as pro-inflammatory and anti-tumor, whereas conversely, M2 macrophages are considered anti-inflammatory and protumor [25,26]. There are a number of subpopulations of M2 macrophages, and there has been a lively academic discussion regarding appropriate nomenclature for the recognized subpopulations [27]. Additionally, there has been a greater understanding from the transcriptome and genomic approach of viewing macrophage activation profiles as a spectrum between M1s and M2s. Consequently, more recent discoveries suggest that TAMs are comprised of multiple distinct subpopulations of overlapping phenotypes, but are generally anti-inflammatory and M2-like [27,28].

Historically, the discovery and categorization of M1 versus M2 was related to their metabolism of arginine [9]. M1 macrophages, via iNOS, preferentially produce nitric oxide, whereas M2 macrophages utilize arginase to produce ornithine [29]. Nitric oxide is a free radical gas that is directly cytotoxic to both cancer cells and a variety of pathogens, underscoring the M1 state as being an activated antitumoral state [9]. In contrast, ornithine is a non-essential amino acid intimately involved in the urea cycle. Though ornithine’s direct role in M2 macrophage production is opaque, elevated ornithine levels have been reported in serum and tissues in a number of malignancies [30]. Additionally, ornithine decarboxylase, a transcriptional target of C-Myc, catalyzes the reaction of ornithine to putrescine and subsequent polyamine synthesis [31]. Polyamines are integral to the DNA stabilization, repair, and cell growth [32,33]. As such, ornithine decarboxylase has been identified as a potential chemotherapy target for several decades, with the goal of inhibiting its downstream production of polyamines [34,35].

Besides arginine metabolism, there are several metabolic pathways and metabolites that influence macrophage polarization [36]. M1 and M2 macrophages have several metabolic differences related to glycolysis, the TCA cycle, and fatty acid metabolism [37]. LPS, which is strongly correlated with an M1 phenotype, increases rates of aerobic glycolysis. Glycolysis facilitates increased carbon flux into the oxidative pentose phosphate pathway, which, in turn, increases the production of reactive oxygen species seen in M1 macrophages. With lower rates of aerobic glycolysis in M2 macrophages, there is higher utilization of the TCA cycle for ATP production. In contrast, M1 macrophages have multiple shunts in the TCA cycle, with the production of various metabolites (e.g., succinate) that are integral to ROS production. Fatty acid oxidation (FAO) is a major energy source for M2 macrophages and polarization. IL-4 increases macrophage FAO, which is also mediated by STAT6 and peroxisome proliferator-activated receptor-gamma (PPARγ) signaling [38].

CD4 T helper cells produce a large subset of immune system cytokines that can be categorized by their downstream effects. Th1 cytokines (e.g., IFNγ) produce a proinflammatory state, whereas Th2 cytokines (IL4,13,10) result in an anti-inflammatory response [20]. An uncontrolled Th1 response will lead to indiscriminate tissue damage, and is thus counteracted by a balanced Th2 response. Regarding macrophages, M1 macrophages stimulate a Th1 immune response, whereas M2 macrophages amplify the Th2 response [39,40]. These Th1 responses are integrated with CD80 and CD86 expression that attracts neutrophils and cytotoxic lymphocytes, which further amplifies M1 macrophage activity. Th2 responses results in growth factor production (TGF-β, VEGF), and further amplifies M2 macrophage activity [9]. In essence, activating either a Th1 or Th2 response can be self-reinforcing for macrophage activity, and lead to a pro-cytotoxic or pro-growth environment.

## 3. Macrophages and Ovarian Cancer

### 3.1. TAMs and Cancer

**Figure 2:**
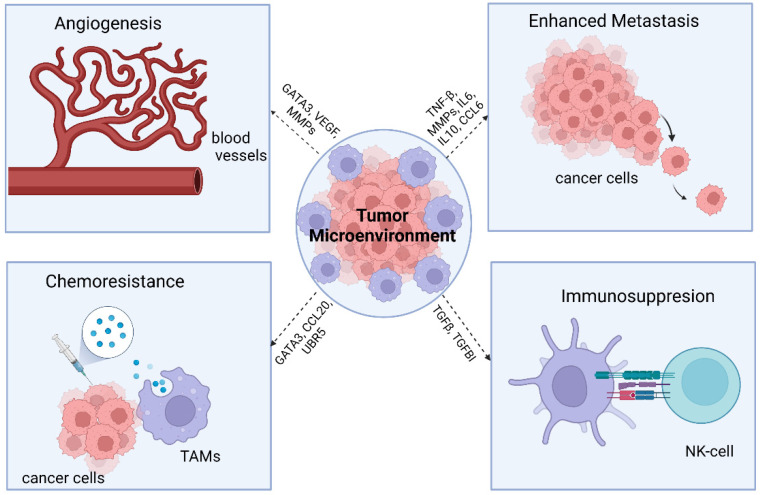
Roles of macrophages within the tumor microenvironment that enhance the progression of malignancy. Created with BioRender.com. Accessed on 21 April 2022.

Prior studies have indicated that high infiltration of TAMs within tumors is associated with adverse clinical outcomes [22] (Figure 2). Additionally, elevated expression of specific cytokines and macrophage chemoattractants within the tumor environment is correlated with a worse prognosis. TAMs have been directly linked to tumor progression via angiogenesis, stromal remodeling, invasion, metastasis, and immune escape [20]. The presence and ratio of M1 to M2 TAMs have been correlated to clinical outcomes in a variety of solid tumors, including, but not limited to, breast, colorectal, gastric, liver, renal, and ovarian [15,22].

A retrospective study analyzing patients with stage III-IV ovarian cancer utilized CD68 and CD163 as M1 and M2 macrophage markers, respectively, to analyze the expression of TAMs within tumor samples. When comparing high vs. low CD163 (M2 TAMs), there was a significant difference in both progression-free and overall survival, both significantly higher in the low CD163 groups [41]. Multivariate analysis also identified the ratio of CD163/CD68 (essentially an M2:M1 ratio) as a negative predictor for overall survival [41]. A meta-analysis of 9 studies, including 794 ovarian cancer patients, demonstrated that a high M1/M2 ratio in tumors predicted an improved prognosis [42].

TAMs originate either from resident tissue macrophages or circulating bone-marrow-derived monocyte precursors [22]. Tissue-resident macrophages are derived from embryonal yolk sac tumors independent of hematopoietic stem cells recruitment [43]. These distinct developmental origins have potential disease implications. In vivo mouse studies of ovarian cancer identified a subset of CD163+ Tim4+ resident macrophages with the omentum that were the primary drivers of metastasis [44]. Transcriptional genetic analysis of ovarian cancer TAMs indicate that TAMs more closely resemble resident peritoneal macrophages than monocyte-derived macrophages [45].

### 3.2. Ovarian Cancer and TAM M2 Macrophage Interaction

TAMs are the predominant immune system cell in the ovarian cancer TME. Data from The Cancer Immunome Atlas (TCIA) and compiled by authors at MD Anderson demonstrate that macrophages make up 39% of the immune cell type in ovarian cancer. This is compared to the second most—CD4 T cells, at 12% (29% for all subclasses of T cells). Of these TAMs, the majority (51%) were M2, compared to 25, 20, and 4% for M0, M1, and monocytes, respectively [46]. These data illustrate that M2 TAMs are the most populous immune system cell in ovarian cancer tumors. 

The interaction between tumor cells and their associated tumor microenvironment is complex and mediated by a number of intracellular signaling pathways (Table 1) [10,22]. Exosomes are a critical mediating factor in the interactions between ovarian cancer cells and the immune system. MiR-200b-containing exosomes released by tumor cells induce an M2 phenotype by downregulating KLF6 in macrophages [47]. Ovarian tumors exosomes containing MiR-222-3p polarized macrophages to an M2 phenotype via an SOCS3/STAT3 pathway [48]. Tumor-associated macrophages (TAM) also release miRNA-containing exosomes which can contribute to disease progression. Multiple miRNA-containing exosomes that were released by TAMs induced an increase in the Treg/Th17 cell ratio via STAT3 inhibition, leading to an immunosuppressive environment [49]. Hypoxia, a common condition within solid tumors including ovarian, increased epithelial ovarian cancer cell release of microRNA-940, which has been shown to induce M2 TAM polarization in vitro [50].

In addition to exosomes, multiple secreted proteins have been found to be involved in cancer cell-mediated M2 polarization. Increased IL-4 signaling from ovarian ID8 cancer cells in mice induced increased PI3K pathway expression, inducing an M2 phenotype [51]. Human epididymis protein 4 (HE4) is known to be upregulated in ovarian cancer cells and correlated with poor clinical prognosis. HE4 has been shown to increase M2 TAM recruitment within malignant ascites in mouse models [52]. CTHRC1 is upregulated in high-grade ovarian cancer, and upregulates STAT6, thus inducing an M2 phenotype [53]. An increased expression of ALOX5AP (arachidonate 5-lipoxygenase activating protein) was associated with both increased M2 macrophages in the tumor microenvironment and a poorer patient prognosis [54].

GATA3, an oncogenic protein, is highly expressed in high-grade serous ovarian cancer (HGSOC) patients. GATA3 is primarily secreted by TAMs, and has been linked to tumor growth, angiogenesis, and chemoresistance [46]. TAMs release epidermal growth factor (EGF) in ovarian cancer, and directly activate the EGFR-ERK pathway [55]. This, in turn, upregulates vascular endothelial growth factor (VEGF) and subsequent angiogenesis [56]. This underscores the role that TAMs play in the proliferation and invasiveness of ovarian cancer.

Metabolic crosstalk between TAMs and ovarian cancer also strongly influences the preponderance of M2 TAMs in the TME. Ovarian cancer cell secretion of hyaluronic acid increases membrane cholesterol efflux from macrophages. In turn, cholesterol upregulates IL-4 secretion and downregulates IFNγ activity via STAT6 and PI3K pathways [51]. Glutamine is considered a key metabolite for cancer cells, yet also has an emerging role in TAM metabolism. In vitro data indicate that ovarian cancer cells release *N*-acetylaspartate, which acts synergistically with IL-10 to polarize macrophages to an M2 with high expression of glutamine synthetase [57].

The interaction between ovarian cancer cells and TAMs is driven by a complex cytokine network that is often mutually reinforcing. For example, ovarian cancer cells are capable of recruiting macrophages via CC-chemokine ligand 2 (CCL2)/Monocyte chemoattractant protein-1 (MCP-1), yet also polarize macrophages to an M2 state [58,59,60]. The studies outlined, amongst others, demonstrate that the ability of ovarian cancer cells to induce an M2 macrophage phenotype is multifaceted and warrants further exploration.

### 3.3. Role of TAMs in Metastasis

Tumor-associated macrophages contribute significantly to metastasis and disease progression [16,61]. In vitro studies have linked macrophage TNF-β secretion with the upregulation of the PI3K/AKT signaling pathways with the promotion of cell migration and invasion [62,63]. TAMs have also been shown to enhance the invasiveness of ovarian cancer cells in co-culture by increasing NF-kb and JNKII activation, thereby inducing the increased release of matrix metalloproteinases (MMP). MMPs, which assist in the degradation of basement membranes, are important early participants in tumor invasion and metastasis [62].

M2 TAMs in coculture with SKOV3 ovarian cancer cells increased STAT3 activation. STAT3 is a critical point of convergence for a number of oncogenic pathways [64]. TAMs also secrete Il-6, which activates the STAT3 pathway, with subsequent enhancement of tumor cell metastasis [65]. Additionally, studies have demonstrated downregulation of the STAT3 pathway with siRNA suppresses ovarian cancer proliferation and induces apoptosis in vitro [66]. STAT3 downregulation also was noted to have decreased expression of VEGF at both the transcription and translation levels [66]. STAT3 overactivation seen in TAMs upregulates IL-10 production [67].

IL-10 is an anti-inflammatory cytokine that directly inhibits the cytotoxic activity of natural killer cells and macrophages [68]. IL-10, in general, is linked to immunosuppressive properties, including reduction of TNF-β expression in TAMs, and enhanced immune system escape [69]. IL-10 also induces immunosuppressive B7-H4 expression on ovarian cancer TAMs [70]. Serum and ascites levels of Il-10 are also correlated to a higher grade and prognosis in ovarian cancer [71,72]. In general, higher serum levels of Il-10, Il-6, and TGF-β are reported in women with advanced ovarian cancer compared to controls [61,73].

TAMs also upregulate TGF-β production via STAT3 upregulation. TGF-β inhibits cytotoxic activity of NK and lymphocytes [74]. TGF-β, secreted by TAMs, increase cancer invasiveness by producing multiple MMPs [75]. MMPs are associated with angiogenesis, enhanced cell migration, and supportive tumor microenvironment roles in ovarian cancer [76]. Other cytokines linked to ovarian tumor progression include insulin growth factor-1 (IGF-1) and CCL18. IGF-1 upregulation is an important signaling pathway in a number of malignancies [77]. High expression of IGF-1 by TAMs induces ovarian cancer cell migration in mouse models [78]. TAMs also secrete CCL18, which is found in high levels of ovarian cancer ascites, and promotes cell migration and metastasis via proline-rich tyrosine kinase 2 and mTOR signaling pathways [79,80].

TAMs play a pivotal role in the transcoelomic (e.g., peritoneal dissemination) spread of ovarian cancer by supporting cell survival and invasiveness [10,16]. TAMs are critical components of ovarian cancer spheroids driving metastasis. TAMs within spheroids have been demonstrated to secrete EGF, leading to downstream upregulation of EGFR and VEGF signaling. Mouse ovarian cancer models treated with erlotinib (an EGFR inhibitor) noted reduced spheroid formation and metastatic progression, underlining an important function of TAMs in disease progression [56]. The milky white patches of the omentum are often one of the first sites of ovarian cancer metastasis—primarily because of their ideal immune microenvironment for cancer cell proliferation. Mice with a depleted milky white patch macrophage population saw a substantial reduction in cancer cell colonization of the omentum. CCL6 secreted by omental macrophages promotes cancer colonization by interaction with CCR1 receptors on cancer cells [81]. The omentum is not only an end site of cancer cell colonization, but also a premetastatic niche that is critical for the development of further metastasis. In fact, malignant ascites was prevalent in mice after omentectomy [44].

TAMs are found in malignant ascitic fluid, and support further ovarian cancer cell migration by releasing tumor growth factor—beta-induced (TGFβI) and tenascin C [82]. TGFβI is an extracellular-matrix (ECM) protein, and dysregulation of the ECM is an important aspect of malignant transformation. Experimental in vitro and mouse models have demonstrated that TAMs are the dominant secretors of TGFBI in both HGSOC tumors and also serous tubal intraepithelial carcinoma (STIC) lesions [83]. STIC lesions are considered a precursor to overt HGSOC [84]. One study compared the TME of the primary tumor site with that of the metastatic site using matched patient samples. Differential gene expression was found at 1468 different genes at the primary versus metastatic TME site, with noted increased signatures involving extracellular matrix remodeling and immune system cell infiltration. High M2 TAM (CD68+/CS163+) presence within the metastatic TME led to a poorer overall survival compared to a lower TAM burden. Intriguingly, the M2 TAM burden did not correlate with the burden of additional infiltrative immune cells: NKp46+, DC-LAMP+, CD20+, and CD8+ cells. These findings strongly suggest TAMs are a major driver of functional immunosuppression in the metastatic TME [85].

In summary, TAMs are instrumental in ovarian cancer disease progression. TAMs function as a powerful driver of immunosuppression within the TME. TAMs also enhance metastasis via multiple signaling pathways, which is highly pertinent in ovarian cancer, a disease often discovered only after metastatic spread. 

## 4. Role of TAMs in Chemoresistance

TAMs play an important role in chemoresistance in ovarian cancer [15,21,61]. Chemotherapy induces phenotypical changes in TAMs. When treated with cisplatin, macrophages in coculture with ovarian cancer cell lines were noted to increased expression in genes related to epithelial-to-mesenchymal transition and cellular stemness. Cancer cellular stemness refers to the capacity of a cell for regeneration and differentiation. Epithelial-to-mesenchymal transition is a crucial first step in cancer metastasis. These findings were demonstrated in platinum-sensitive, but not resistant, lines, although there was a repolarization to an M2-state in both sensitive and resistant lines [86]. Additional co-culture experiments of ovarian cancer cells with macrophages demonstrate induced cellular stemness via IL-8/STAT3 signaling [87]. Platinum-agents have demonstrated increased IL-6 and PGE2 production in ovarian cancer cell lines, with subsequent activation of STAT3 pathway and induction of M2 polarization with upregulation of IL-10 [88]. Thus, standard platinum agents may actually increase the underlying immunosuppressive tumor microenvironment by driving M2 TAM polarization, leading to indirect chemoresistance. Cisplatin has also been shown to stimulate “classically-activated macrophages” (i.e., M1) to increase CCL20 cytokine production and enhance ovarian cancer cell migration via CCR6 [89]. The CCL20-CCR6 axis has been linked to enhanced metastasis in a variety of malignancies, including ovarian [90]. Additionally, CCL20 has been associated with paclitaxel resistance in subgroups of ovarian cancer cell lines [91].

Ubiquitin protein ligase E3 component n-recognin 5 (UBR5) is an E3 ligase that plays a role in platinum resistance in ovarian cancer [92]. Additionally, UBR5 expression has been correlated with worse prognosis and survival outcomes in ovarian cancer [93]. In vivo models demonstrate UBR5 mediates TAM recruitment and infiltration into ovarian tumors via the CCL2/CSF-1 axis. UBR5 knockout models demonstrate attenuated tumor growth and greater sensitivity to platinum chemotherapeutics [94].

In vitro, apoptotic ovarian cancer cells induce macrophages to polarize to the M2 phenotype. Troublingly, this suggests that apoptotic cells induced by chemotherapy may contribute to the enhanced formation of M2 macrophages, and a further immunosuppressive TME [95]. Different drug regimens have different effects on supporting either a pro-inflammatory or immunosuppressive TME. The most used ovarian cancer regimen of carboplatin and paclitaxel was found to induce a proinflammatory state by upregulation of IFNγ. Contrarily, a regimen of carboplatin and gemcitabine induced an immunosuppressive environment [96]. Independently, paclitaxel has been shown to induce a M2 to M1 repolarization in vivo via TLR4. Gene expression analysis on biopsies performed before and after three cycles of paclitaxel monotherapy in ovarian cancer patients demonstrated upregulation of M1 macrophage genes [97].

Platinum-based chemotherapy remains a cornerstone of ovarian cancer treatment, with the development of platinum resistance as a dominant prognostic marker [98]. Evidence suggests that TAMs play an essential role in inducing chemoresistance via both direct (e.g., URB5 expression) and indirect mechanisms (STAT3 signaling).

## 5. TAM Immunotherapy

Immunotherapies have successfully treated a variety of diseases, including certain cancers, for decades [99]. Ovarian cancer is a heterogeneous disease, but specific immunologic biomarkers have displayed prognostic significance. A meta-analysis including ten studies demonstrated that the absence of tumor-infiltrating T cells significantly worsened survival outcomes (pooled HR: 2.24, 95% CI; 1.71–2.91) [100]. Despite evidence of a highly active immune tumor environment, the field of immunotherapy has experienced relatively limited success in treating ovarian cancer [101]. Immunotherapy strategies that have demonstrated success in other gynecological malignancies, such as checkpoint inhibition (e.g., PD-L1 blockade) [102], have displayed underwhelming results in ovarian cancer treatment [103,104]. A primary hypothesis for this lack of immunotherapy success is the highly suppressive ovarian cancer tumor microenvironment resulting in a “cold” immune phenotype [105,106,107].

One historical approach to overcome the immunosuppressive TME was the systemic administration of immune-activating cytokines. IFNγ polarizes macrophages to an M1 state, and prior studies demonstrated early success with this approach, including administration of intraperitoneal IFNγ at second-look laparotomy [108]. Another phase III trial utilizing IFNγ 0.1 mg subcutaneously on days 1, 3, 5, 15, 17, and 19 of each 28-day cycle + 100 mg m^−2^ cisplatin and 600 mg m^−2^ cyclophosphamide in the upfront adjuvant setting demonstrated an improvement in 3 year PFS (51% experimental arm vs. 38% control, *p* = 0.031). Toxicity was deemed acceptable, though noted a near-ubiquitous flu-like syndrome in the experimental arm [109]. However, recruitment was terminated early due to significant changes in the primary treatment of ovarian cancer [109]. A final phase III trial, performed with the new standard of care of carboplatin/paclitaxel, was stopped at the second interim analysis due to significantly worse survival outcomes in the experimental IFNγ arm (OS: 1138 days vs. not estimable, HR = 1.45, 95% CI = 1.15–1.83). Additionally, there were considerably higher significant adverse events in the experimental arm (48.5% vs. 35.4%). As IFNγ is a non-specific and powerful immune cytokine, it was postulated that there was paradoxical immune suppression related to chronic administration [110].

**Figure 3:**
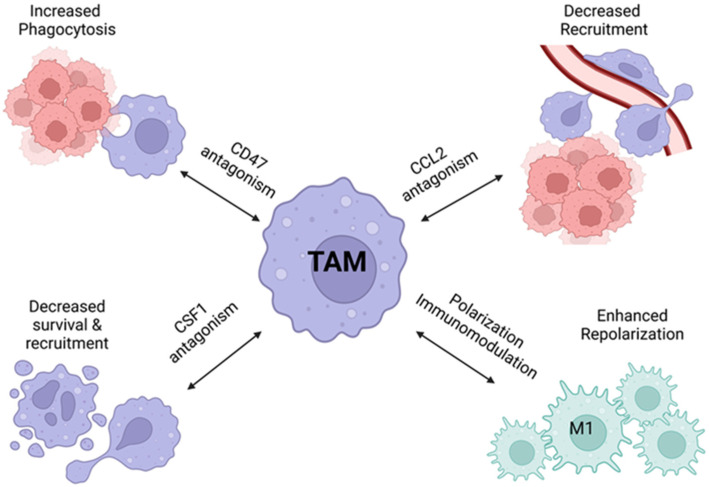
Anti-TAM therapeutic strategies. Created with BioRender.com. Accessed on 14 March 2022.

Precision therapeutics that target TAMs are a proposed strategy at overcoming the cold immunophenotype TME. Early-phase clinical trials are underway exploring various macrophage-centric approaches, including targeting M2 macrophages directly via CSF-1R pathway, anti-CD47 antibodies to enhance macrophage phagocytosis, and anti-CCL2 antibodies to decrease macrophage recruitment [81] (Figure 3). CC-chemokine ligand 2 (CCL2)/Monocyte chemoattractant protein-1 (MCP-1) has been shown to mediate TAM recruitment, and indirectly promote tumor growth and progression [111]. Serum CCL2 has elevated levels in ovarian cancer patients [112]. Pre-clinical data have investigated bromodomain and extra-terminal domain inhibitors (BETi) targeting the CCL2/CCR2 axis, with promising in vitro and in vivo results. Additionally, BETi therapy was shown to enhance the efficacy of anti-angiogenesis therapy (e.g., bevacizumab) in murine models [113]. A phase I trial of carlumab, a CCL2 monoclonal antibody, in solid tumor patients, included eight ovarian cancer patients, and 1/8 of patients had a stable disease at 10.5 months [111].

There are several pre-clinical and early clinical trials investigating the restoration of phagocytosis in TAMs via the inhibition of the CD47/SIRPa pathway [114,115]. CD47 is a marker of self-identification, and prevents phagocytosis by macrophages in the TME and as a transmembrane protein, and functions as a marker for inhibitor of macrophage phagocytosis (“don’t eat me signal”) [116,117]. CD47 is highly expressed in ovarian cancer, and has been linked to worse clinical prognosis, including progression-free survival [118]. In vitro studies exploring anti-CD47 therapy in ovarian cancer report the restoration of macrophage phagocytosis [119]. Early-phase clinical trials have shown limited efficacy. In a phase I trial utilizing Hu5F9-G4, a CD47 antibody, 13 of 62 participants had advanced ovarian cancer. Two of these patients with ovarian cancer had partial responses lasting for 5.2–9.2 months [115].

As mentioned previously, the CSF-1/CSF-1R axis is the primary regulator of macrophage recruitment and differentiation. Due to its cornerstone role in TAM development, there has been considerable interest in targeting the axis for cancer therapy [120,121]. Pre-clinical studies investigating GW2580, a CSF-1R inhibitor, demonstrated decreased tumor volume, ascites, and TAM infiltration in ovarian cancer mouse models [122]. Intriguingly, there was also a demonstrated repolarization of macrophages to an M1 state with targeting CSF-1 and an increased favorable CD8/CD4 T cell ratio [122]. This raises the specter of macrophage repolarization as a therapeutic stratagem. Several early clinical trials have also investigated the inhibition of CSF-1 or CSF-1R. Investigations of CSF-1R antibodies in patients with advanced solid tumors include: 1) AMG 820 [123], emactuzumab, in monotherapy or plus paclitaxel [124], and LY3022855, plus durvalumab or tremelimumab [125], each displaying modest activity in ovarian cancer patient subsets. Another phase 1b study evaluated Pexidartinib, a CSF-1R tyrosine kinase inhibitor, plus paclitaxel, in advanced solid tumors. Of six patients with platinum-resistant or refractory ovarian cancer, one displayed a complete response, and one a partial response. It also displayed modest activity in ovarian cancer [126].

Overall, the recent clinical evidence of TAM therapeutic strategies, such as CSF-1R inhibition and CD47 targeting, has been underwhelming (Table 2). Additionally, attempts at global macrophage attenuation via IFNγ administration were unsuccessful in phase III clinical trials. TAMs do display immune checkpoint modulators, such as PD-L1 [16], and may respond to anti-PD-L1 therapy, even if not the primary target [81]. Regardless, anti-PD-L1 clinical trials failed to demonstrate any significant survival advantage in ovarian cancer patients [106]. Though TAMs remain promising targets for ovarian cancer immunotherapy, alternative strategies are required. 

## 6. Pharmacological Modulation of TAMs and Ovarian Cancer

An additional therapeutic framework with promise is a strategy for repolarizing protumor M2 macrophages to a tumoricidal M1 phenotype [127]. A higher M1/M2 ratio in the tumor microenvironment correlates with better survival outcomes in ovarian cancer patient; thus, a “re-education” of macrophages to an antitumor phenotype holds theoretical promise [128]. Multiple methods of repolarizing M2 TAMs have been explored, and with the goal of precision targeting and limited off-target effects.

Toll-like receptor (TLR) 7/8 agonists are immunostimulatory therapeutics with the capacity to repolarize macrophages to an M1 state. However, TLR 7/8 agonists have poor pharmacokinetic profiles and significant off-target effects. Large, anionic liposomes loaded with resiquimod, a TLR 7/8 agonist, were able to target and repolarize TAMs to an M1 state when administered intraperitoneally in murine ovarian cancer models [129]. Additional TLR 7/8 agonist, imidazoquinoline IMDQ, was conjugated with a nanobody (a fragment of the antigen-recognizing region of the antibody heavy chain) to target only the TAMs of interest, not a global macrophage response. The nanobody targeted the macrophage mannose receptor, a surface marker of immunosuppressive TAMs, making it an ideal molecule for limiting off-target effects. This imidazoquinolinone-nanobody conjugate was able to successfully reprogram M2 macrophages to an M1 phenotype in ovarian cancer mouse models [130].

Multiple groups have taken an interest in the role of NF-kβ in the immune targeting of cancer cells. [131] The NF-kβ signaling pathway has been described as a major regulator of ovarian cancer progression and chemoresistance [132]. IKKβ is an upstream activator of NF-kβ, and STAT6 is a transcription factor necessary for M2 polarization. When using a combinatorial IKKβ siRNA and a STAT6 inhibitor delivered inside a pH-sensitive micellular compound, there was demonstrated M2 to M1 repolarization both in vivo and in vitro [133]. Methotrexate coupled dendron nanoparticle displayed TAM depletion in murine ovarian cancer models, in addition to reducing angiogenesis and cancer stem cells [134].

More targeted approaches to modulation have demonstrated promise. Bromodomain containing 4 (BRD4) protein, an epigenetic regulator, has been associated with M2 to M1 polarization [135]. BRD4 interacts with acetylated histone tails and supports the transcription of oncogenic genes [136]. In vitro studies with AZD5153, a novel BRD4 inhibitor, demonstrated repolarization of M2 macrophages to M1 and activation of CD8+ T lymphocytes, while also sensitizing ovarian cancer cell lines to anti-PD-L1 antibodies [137]. Nanoparticle TAM targeting has also demonstrated feasibility against TAMs in both in vivo and in vitro models. Silica and polylactic-***co***-glycolic acid-based anionic nanoparticles have shown selective accumulation in ovarian tumor TAM mouse models [138]. Encapsulation of miR-125b, an mRNA within hyaluronic acid nanoparticles, demonstrated repolarization of M2 macrophages to M1 in malignant ascites in an ovarian cancer mouse model [139].

## 7. Future Directions

Clodronate is a bisphosphate with current clinical use in the treatment of osteoporosis, Paget’s disease, hypercalcemia, multiple myeloma, and breast cancer [140]. Bisphosphonates’ primary clinical use are via the inhibition of osteoclast bone resorption, but experimentally, liposomal clodronate is used for macrophage depletion [140,141]. Bisphosphonates are used in the adjuvant setting in early-stage breast cancer, as they reduce bone recurrences and improve survival outcomes [142,143]. Bisphosphonates also reduce pathological fractures and skeletal-related events in multiple myeloma [144]. When clodronate is encapsulated in liposomes, there is preferential phagocytosis by macrophages, resulting in a global macrophage depletion [145]. In vivo studies have demonstrated that liposomal clodronate inhibits angiogenesis and tumor progression in ovarian cancer mouse models [146,147]. There are currently no active clinical trials utilizing liposomal clodronate in cancer therapy, despite promising results from in vivo and in vitro data. One drawback is the non-specific “global” macrophage depletion and potential for resultant adverse off-target effects [148]. However, groups are investigating novel liposomal formulations with greater cell specificity [149].

Immune-cell-derived exosomes have been proposed as TAM repolarization therapeutics [146]. Exosomes, which are membrane encased vesicles, can be derived from various cell types, and are intricately involved in cellular signaling [150]. Exosomes are promising due to their endogenous characteristics enabling immune system evasion and the ability to be loaded with a variety of therapeutic cargos [151]. Prior research has demonstrated that macrophage-derived exosomes can repolarize TAMs in vitro and effectively target tumors in vivo [152,153]. Exosomes derived from murine M1-polarized macrophages were able to repolarize M2 TAMs to an M1 state in vitro and in vivo using a murine colorectal carcinoma model [154]. To date, there has been limited exploration into this strategy for ovarian cancer, but it remains an intriguing option.

## 8. Conclusions

Ovarian cancer is a highly lethal female malignancy that is unresponsive to current immunotherapy approaches. A major reason for this cold immunophenotype is the highly immunosuppressive TME, which, in turn, is dominated by TAMs. TAMs have been directly linked with increased tumor invasiveness, enhanced metastasis, angiogenesis, chemoresistance, and worse clinical survival outcomes. Early clinical TAM-specific therapeutic strategies, including increasing macrophage phagocytosis and restricting recruitment, have been underwhelming. However, macrophage phenotype modulation, in which protumor M2 TAMs are repolarized to an antitumor M1 state, is a promising strategy, and warrants further investigation.

## Figures and Tables

**Table 1:** Factors influencing M2 Induction.

Factor	Mechanism
miR-200b exosomes	Downregulation of macrophage KLF6
miRNA exosomes (released from TAMs)	Upregulation of CD4+ Treg and Th17 cells
IL-4	Increased PI3K signaling
Human epididymis protein (HE4)	Increased M2 recruitment
IL-4 and IL-13	STAT6 upregulation
Hypoxia	microRNA-940

**Table 2:** Anti-TAM Therapeutic Clinical Trials.

Phase	Drug	Mechanism	Study Population	Results	Study Citation
III	IFNγ	M2 to M1 polarization	Ovarian cancer	OS: 1138 days vs. not estimable, HR = 1.45, 95% CI = 1.15–1.83; favoring control arm Higher significant adverse events in the experimental arm compared to control (48.5% vs. 35.4%).	[103]
I	Carlumab	CCL2 monoclonal antibody	Advanced solid tumors	Subset: Eight ovarian cancer patients, 1/8 of patients had a stable disease at 10.5 months	[104]
I	Hu5F9-G4	CD47 antibody	Advanced solid tumors	Subset: Thirteen ovarian cancer patients, 2/13 with partial responses lasting for 5.2–9.2 months	[108]
I	AMG 820	CSF-1R antibody	Advanced solid tumors	Subset: Two ovarian cancer patients; none available for tumor response	[116]
I	Emactuzumab +/− paclitaxel	CSF-1R antibody	Advanced solid tumors	Subset: monotherapy (15 ovarian cancer patients)—0/15 with response, combination (13 ovarian cancer patients)—1/13 with partial response	[117]
I	Pexidartinib + paclitaxel	CSF-1R tyrosine kinase inhibitor	Advanced solid tumors	Subset: Six ovarian cancer patients: 1/6 with complete response and response duration 189 days, 1/6 with partial response and response duration 94 days	[119]
